# Can We Talk through a Robot As if Face-to-Face? Long-Term Fieldwork Using Teleoperated Robot for Seniors with Alzheimer's Disease

**DOI:** 10.3389/fpsyg.2016.01066

**Published:** 2016-07-19

**Authors:** Kaiko Kuwamura, Shuichi Nishio, Shinichi Sato

**Affiliations:** ^1^Graduate School of Engineering Science, Osaka UniversityOsaka, Japan; ^2^Hiroshi Ishiguro Laboratory, Advanced Telecommunications Research Institute InternationalKeihanna Science City, Kyoto, Japan; ^3^Graduate School of Human Sciences, Osaka UniversityOsaka, Japan

**Keywords:** elderly care robot, teleoperated robot, Alzheimer's disease, elderly care facility, gerontology

## Abstract

This work presents a case study on fieldwork in a group home for the elderly with dementia using a teleoperated robot called Telenoid. We compared Telenoid-mediated and face-to-face conditions with three residents with Alzheimer's disease (AD). The result indicates that two of the three residents with moderate AD showed a positive reaction to Telenoid. Both became less nervous while communicating with Telenoid from the time they were first introduced to it. Moreover, they started to use more body gestures in the face-to-face condition and more physical interactions in the Telenoid-mediated condition. In this work, we present all the results and discuss the possibilities of using Telenoid as a tool to provide opportunities for seniors to communicate over the long term.

## 1. Introduction

**Figure 1:**
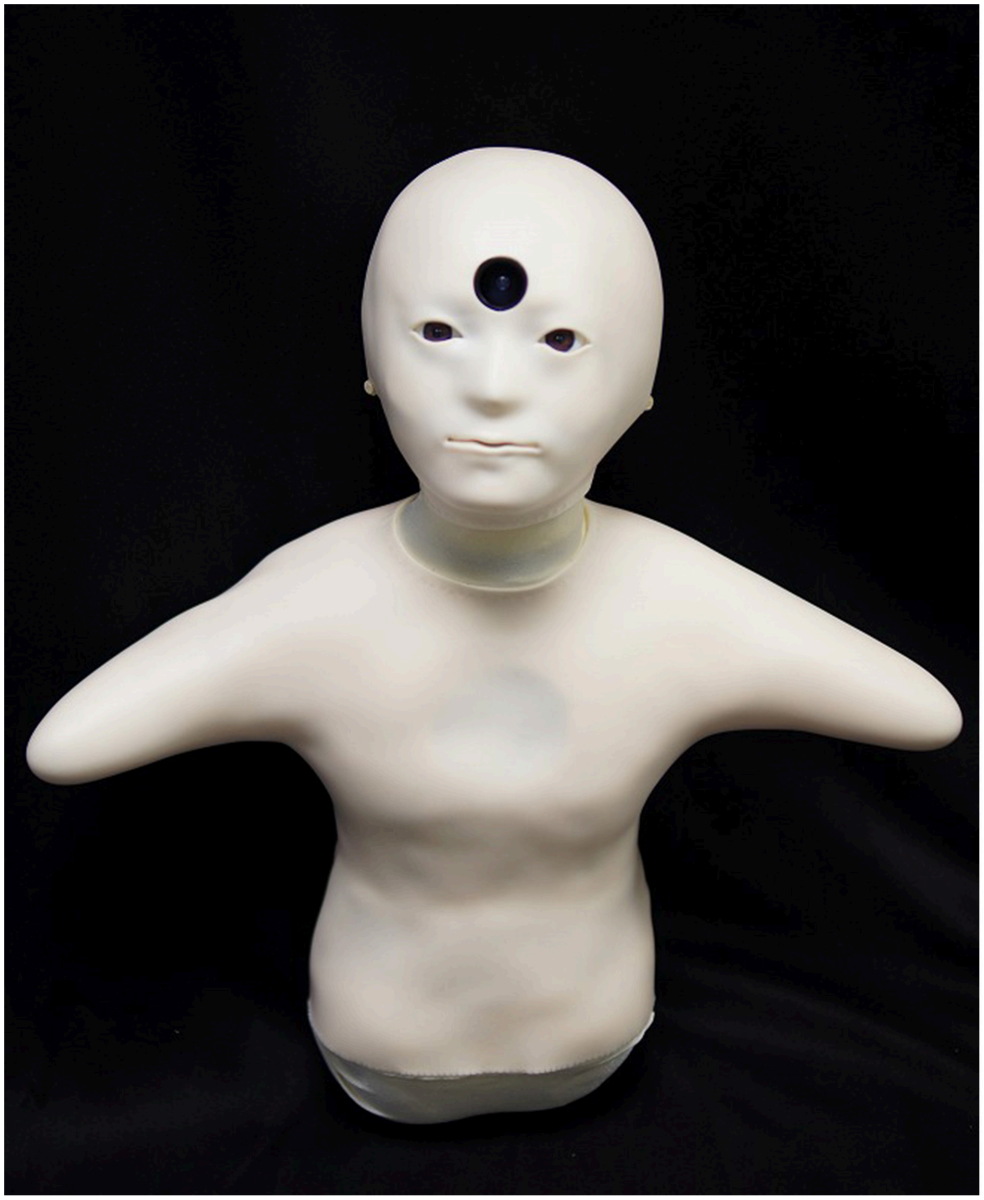
**Telenoid R3b**.

This work presents a case study on fieldwork in a group home for the elderly with dementia using a teleoperated robot. We developed a robot called Telenoid to provide communication support for seniors (Figure 1). Telenoid is a teleoperated robot covered with soft vinyl that can transmit a remote operator's physical movements and voice. Telenoid users can physically interact (hug and touch) with the robot while communicating with an operator who can communicate from a remote place through the Internet. From experiments in Japan and Denmark, we found that seniors quickly became fond of interaction with Telenoid, and seniors with dementia also liked it (Yamazaki et al., 2014). However, the effects of using it and how communication differs when talking through Telenoid compared to face-to-face communication are not clear.

In this paper, we describe a long-term fieldwork conducted in a group home (a community-based care facility where mild/moderate demented seniors live together) and compared face-to-face communication with communication mediated through Telenoid. We discuss the possibilities of using Telenoid as a tool to support long-term communication between people and elderly individuals.

### 1.1. Background

The population of senior citizens is rapidly increasing worldwide. In Japan, more than a quarter of the population is already over 65 (Cabinet Office, Government of Japan, 2014). The number of elderly with dementia has reached 4.6 million, and an additional 4 million people probably suffer from mild cognitive impairment (MCI). The Japanese Ministry of Health, Labor and Welfare estimates that the social cost of elderly with dementia was 14.5 trillion yen (approximately 118 billion US dollars) in 2014.

This trend, which is not specific to Japan, can also be seen globally (United Nations, 2013). In the more developed regions, populations aged 60 or over are expected to increase by 45% from 287 million in 2013 to 417 million in 2050. In the less developed regions, populations aged 60 or over are currently increasing even faster, and the numbers are expected to rise from 554 million in 2013 to 1.6 billion in 2050. With an increase of senior citizens, the number of people suffering from dementia is also likely to rise and will impose a severe social cost.

As societies continue to age, the number of seniors living alone will increase. Such changes limit opportunities to communicate with others and weaken their connection to society. Such limited society connections increase the risk of dementia (Fratiglioni et al., 2000). Furthermore, as the degrees of dementia progress, seniors become more withdrawn and experience more difficulty communicating with others including caregivers.

The most common cause of dementia is Alzheimer's disease (AD), which is perhaps responsible for up to 60–70% of all dementia cases (World Health Organization, 2015). AD is a chronic progressive neurodegenerative disorder characterized by the following symptoms: memory loss, language difficulty, executive dysfunction, psychiatric symptoms, such behavioral disturbances as depression, hallucinations, delusions, agitation, and difficulty performing daily living activities (Burns and Iliffe, 2009). Seniors with AD sometimes reject care and become depressed or belligerent as a result of the behavioral and psychological symptoms of dementia (BPSD). They forget what they have done or said in the short term due to memory impairment. Understanding both the physical and mental conditions of seniors is important for taking care of them. However, accurately determining their mental conditions is difficult since identifying clues that might elucidate their emotional states when they are depressed are complicated. Therefore, it is important for caregivers to motivate seniors with AD to communicate to cope with BPSD and to suppress progress of dementia.

At the same time, the aging of society is exacerbating caregiver shortages. In fact, the lack of caregivers and their job turnover is already severe in both developed and developing countries (Kingma, 2007). According to a survey by a careworker foundation in Japan, 59.3% of caregivers feel overworked due to the actual lack of caregivers whose annual turnover rate has reached 16.5% (Care Work Foundation, 2015). Although the number of seniors who need care is increasing, the number of people who work as caregivers is decreasing, due to low wages (61.3%) and physically/mentally hard work (49.3%). Improving caregivers' working lives and motivating them is crucial (Lu et al., 2012).

The lack of caregivers makes caregivers busy and decreases opportunities for caregivers to communicate with residents. If seniors suffer from severe AD, they rarely respond to care. As a result, caregivers have difficulty communicating with their charges and become discouraged. To maintain their motivation, caregivers need skills and adequate time to properly communicate with seniors with dementia. However, this requires experience and training, and it is especially difficult for new/inexperienced caregivers who are often too busy to take time to communicate with their residents.

In Japan, there are volunteers who visit care facilities periodically to have conversation with residents. For smooth communication with the residents with AD, the volunteers need to be trained. Even though they provide opportunities for seniors to have conversation, they cannot attend the facilities every day. The volunteers usually belong to non-profit organizations and can only visit facilities near their houses occasionally. In the facility at which we conducted our experiment, volunteers only visit once or twice a month and talk with just a limited number of residents. Although there are telephones in houses or care facilities, residents with AD rarely use it to have conversation with others. This may be partially due to their weakened hearing ability by aging but also due to their lack of motivation to speak with others. With the progress of AD, one feels difficulty in composing and understanding dialogue properly. By recognizing the decline in their ability, residents with AD quickly lose their motivation to speak with others.

In this paper, we introduce a teleoperated robot Telenoid, which can be teleoperated from remote place. By using Telenoid, seniors living alone or in nursing homes will have more opportunities to communicate with their family or volunteers. The small and soft body of Telenoid allows people to hold it while having conversation through it, allowing one to have communication with multiple modalities including visual and tactile sensations besides dialogue. Moreover, Telenoid's child-like appearance might attract residents and motivates them to communicate. If Telenoid can motivate residents to communicate, they will become more active or emotional, and caregivers will be able to understand their physical and mental conditions easier.

### 1.2. Related works

Recently some attempts have started using information technologies and robots to increase the opportunities for seniors to communicate. One example is the Mobile Robotic Telepresence (MRP) system, which is a video conferencing system mounted on a mobile robotic base. It allows users to telecommunicate with residents from remote locations, and several researches have been carried out with it (Beer and Takayama, 2011; Orha and Oniga, 2012; Kristoffersson et al., 2013). Kuwahara et al. (2006) developed networked reminiscence therapy, which effectively increases the self-esteem of and reduces the behavioral disturbances in seniors with dementia (Kuwahara et al., 2006). Their system combines IP video phones with a photo- and video-sharing facility. In their experimental results, elderly with dementia communicated with therapists by videophone, and networked reminiscence sessions were generally as successful for individuals with dementia as face-to-face reminiscence sessions. We also tried to introduce tablets and video chat to the residents who showed interested in such new devices. However, they soon returned them to us. Although they seemed willing to directly communicate with others, they were discouraged from using such communication tools as phones or video chat. We believe that to increase the opportunities for seniors to communicate, it is important to not just introduce a communication device but also to motivate them to use it.

Perhaps the most famous elderly care companion robot is Paro, a baby seal robot designed for therapy (Wada et al., 2005). It has sensors on its body and reacts with sound and several actuators. Its cute appearance and behavior stimulates the interest of the elderly. Compared to the resident dog, the residents who interacted with Paro significantly felt less loneliness, and they also talked to it and touched it more than the resident dog (Robinson et al., 2013). From seniors with mild/moderate dementia, Paro evokes natural expressions more frequently than stuffed animals and is likely to increase the willingness of the staff members to communicate and work with elderly people with dementia (Takayanagi et al., 2014). However, since it is not designed for verbal communication, seniors talk to Paro, which reacts but cannot have a conversation.

To introduce a robot to elderly care houses, caregivers must constantly use it and residents must be discouraged from losing interest in it. Manuals for use and introduction in care facilities exist for Paro (Wada et al., 2010), and Kanagawa Prefecture in Japan also provides support for introducing robots into care facilities. These allow users to properly employ such robots; otherwise, users will lose interest and stop using them. Tanaka et al. (2007) updated the behavior of a robot called QRIO during trials to maintain the interest of a classroom of toddlers. Otherwise, children seldom reacted to it. Users might lose interest in robot because of low intelligence, or few variety of reaction in the robot. Sabelli et al. (2011) placed a robot called Robovie2, which was remotely controlled by an operator, in an elderly care center for 3.5 months. Through the ethnographic study, they found that the robot was accepted in the community. However, they provided only ethnographical descriptions and performed no statistical data analysis. As such, although there have been trials to use robots in care facilities for rather long duration, study with objective measurements have been missing and effective methodologies for utilizing robots while keeping people's interested have been unclear.

From experiments of Telenoid in Japan and Denmark, we found that seniors with dementia often showed strong attachment to and liked to communicate with Telenoid (Yamazaki et al., 2014). Although it is difficult to communicate with seniors with dementia, school children were able to communicate with the residents without training by using Telenoid (Yamazaki et al., 2013). We found that Telenoid could motivates seniors with dementia to have conversation with others, while making people talking through Telenoid to be much relaxed compared to face-to-face. However, the quality of the conversation and how third person such as caregivers observing the interaction feels are unrevealed. Also, how people's response to Telenoid changes in longer term is not clear.

In this paper, we described a long-term fieldwork conducted in a group home (a community-based care facility where mild/moderate demented seniors live together) and compared face-to-face communication with communication mediated through Telenoid. We evaluated the quality of the conversation by questionnaire. The questionnaire was answered by the speaker and the observer to reveal the effect of third person. We discussed the possibilities of using Telenoid as a tool to support long-term communication between people and elderly individuals.

## 2. Methods

### 2.1. Participants and ethics statement

Three female residents (from 85 to 96 years old) of a senior group home participated in this study. They were all clinically diagnosed as AD. Informed consent was obtained from the group home manager, the doctor in charge, and the participant families. This experiment was approved by the Human Ethics Committee of the Graduate School of Human Sciences, Osaka University (No. 26-60), and the Ethics Committee of the Advanced Telecommunications Research Institute International (No. 14-602-3).

### 2.2. Procedure

The experiments were conducted once or twice a week for 3 months in a group home for seniors with dementia in Osaka, Japan. The dates and times of the trials were adjusted based on the conditions of the participants and the convenience of the group home. All conversations were exchanged in a public space, either in the dining room or the TV room.

Participants spoke with a person (henceforth *speaker*) in a face-to-face condition (Face condition) and a Telenoid-mediated condition (Telenoid condition). The conditions were randomly ordered and the duration of the conversations was limited to 15 min each. The conversations were suspended when the participant was not feeling well or was unwilling to talk. An *observer* monitored the interaction between the participant and the speaker in both conditions. After both conditions were conducted, the speaker and the observer answered questionnaires. We recruited five university students who major in gerontology as evaluators. None of the evaluators had experience of using robots. They played the speaker and observer roles in turn. We asked them to make evaluation in the quality of conversation and made no further specific instructions.

**Figure 2:**
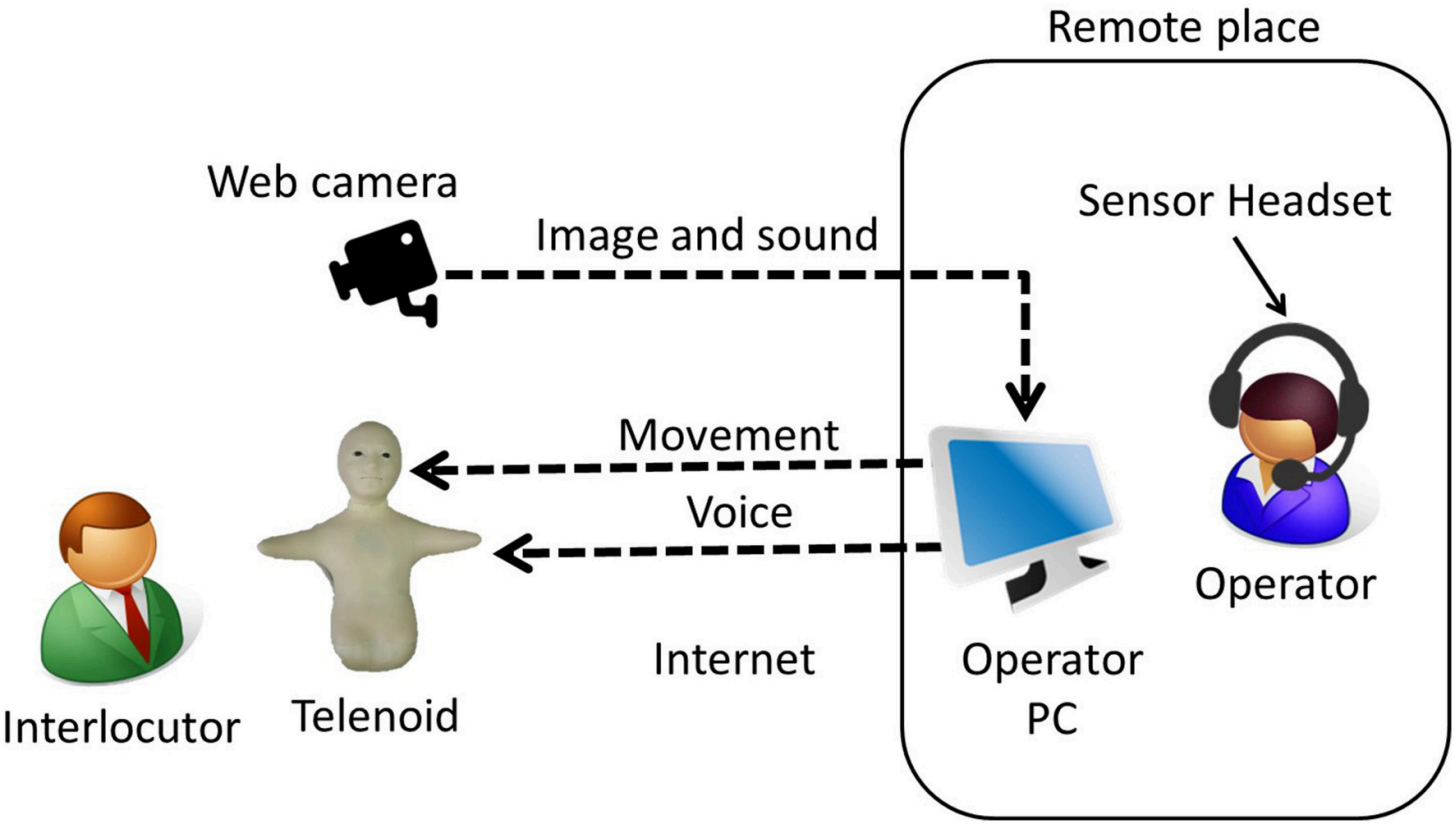
**Teleoperating system**.

In the Telenoid condition, the speakers controlled a Telenoid R3b (Figure 1) to communicate with the elderly participants by a teleoperation system from a remote location (Figure 2). Another experimenter first carried Telenoid and sat in front of the participant. During the conversation, the experimenter gave Telenoid to the participant, and if the participant did not refuse it, the participant held it and continued the conversation. When participants held Telenoid, they put it on their laps and sometimes leaned it against a desk. Telenoid has six independent actuators (jaw movement, yaw, pitch, and roll movement for its neck and horizontal movements for each arm) that allow it to synchronize motion with the speaker. The speaker's head motion is captured by sensors (three-axis accelerometer and three-axis magnetometer) embedded in a headset and transmitted to the robot. Speech-driven lip motion generation, which creates lip motions from the speaker's vocal information, is used to control Telenoid's jaw movement (Ishi et al., 2011).

### 2.3. Evaluation

#### 2.3.1. Diagnosis of dementia

The caregivers of the group home answered the following cognitive function tests before and after the experiment. We used these tests to measure the cognitive function of the participants and AD's progress during the experiment.

1. Mini-Mental State Examination (MMSE): 30-point questionnaire that is used extensively in clinical and research settings to measure cognitive impairment (Pangman et al., 2000). Any score greater than or equal to 27 points (out of 30) indicates normal cognition. Scores below indicate severe (≤ 9 points), moderate (10–18 points), or mild (19–24 points) cognitive impairment (Mungas, 1991).
2. Quality of life questionnaire for dementia (QOL-D): 31 items grouped into six response sets to measure six domains of health-related QOL (Terada et al., 2002).
3. Dementia Behavior Disturbance Scale (DBD): 28 items, measured by the frequency of BPSD on a five-point scale (Mizoguchi et al., 1993).
4. Japanese version of the Neuropsychiatric Inventory (NPI-NH): measures 12 symptoms of neuropsychiatric disturbances (Hirono et al., 1997).
5. Barthel Index (BI): measures performances of activities of daily living (ADL) by 10 items (Shah et al., 1989). A total BI score of 0–20 suggests complete dependence, 21–60 indicates severe dependence, 61–90 indicates moderate dependence, and 91–99 indicates slight dependence.
6. Vitality Index (VTI): measures vitality related to ADL in elderly patients with dementia by five subscales (Toba et al., 2002).

#### 2.3.2. Questionnaire

The speaker and the observer filled out the following questionnaire, where each item was rated on a five-point scale:

Q1 Smoothness of conversation (rough-smooth)
Q2 Amount of conversation (poor-rich)
Q3 Quality of conversation (low-high)
Q4 Impression of participant (gloomy-cheerful)
Q5 Emotional state of speaker (nervous-relaxed)
Q6 Emotional expression of speaker (poor-rich)
Q7 Understanding participant (not understood-understood)

Items in the questionnaire were listed to measure the quality of the conversation. Q1–3 measures the quality of the conversation more quantitative, and Q4–7 measures the impression of the residents and speaker more qualitative. We included these items to measure whether residents were motivated to communicate, and to measure the impression of observer observing the conversation.

Hereafter, we denote a speaker's response to Qn as Sp_Qn and an observer's response to Qn as Ob_Qn. The questionnaire scores were compared between the Telenoid and Face conditions within subjects by paired *t*-tests to reveal the effect of using Telenoid. We compared the scores of the first and last five trials in each condition (by Student's *t*-test when homoscedasticity was confirmed and Welch's *t*-test when unconfirmed) to determine any long-term effects.

#### 2.3.3. Video analysis

A surveillance camera in each room (the dining and TV rooms) and one mobile camera were used to record the interactions. From the video recordings, we counted the number of times that the participants used body gestures and made physical contact. Due to limited views, we counted only the number of clear upper body gestures and physical contacts. For control between the Telenoid and Face conditions, hugs in the Telenoid condition were excluded from gestures and physical contacts.

We used a paired *t*-test between the Telenoid and Face conditions within subjects to reveal the behavioral differences using Telenoid. We also compared the frequency of such behaviors of the first and last five trials in each condition (by Student's *t*-test when homoscedasticity was confirmed and Welch's *t*-test when unconfirmed) to determine the long-term effect.

## 3. Results

We conducted 10 trials (interactions) for each participant. The average duration of an interaction was 709.1 s (*SD* = 316.2) for the Face condition and 798.7 s (*SD* = 383.3) for the Telenoid condition. The Telenoid condition time was longer because residents kept talking to Telenoid even after they were informed of the experiment's end.

### 3.1. Ms. A: 96 years old

#### 3.1.1. Diagnosis of dementia

Ms. A was diagnosed as AD in 2006. The test results for the diagnosis of dementia before and after the experiment are shown in Table 1.

**Table 1:** **Diagnosis of dementia test result of Ms. A**.

		**Before (11/13/2014)**	**After (3/26/2015)**
MMSE		12/30	13/30
QOL-D	Positive affect	28/28	28/28
	Negative affect and actions	8/24	7/24
	Ability of communication	20/20	20/20
	Restlessness	8/20	7/20
	Attachment with others	10/16	14/16
	Spontaneity and activity	14/16	13/16
DBD		13/112: No major problem in	8/112: No major problem in
		mental and behavioral disorder	mental and behavioral disorder
NPI-NH		Agitation/aggression	None
		Frequency 1, severity 1, caregiver distress 1	
BI		45/100	45/100
VTI		8/10	8/10

Her MMSE score were 12 (before) and 13 (after), indicating that Ms. A had moderate dementia. However, BPSD, which was previously observed when she was staying at home and in another geriatric health service facility, did not appear in the current group home. During the experiment, an episodic memory disorder was discovered in Ms. A. No other remarkable cognitive impairments were found.

We conducted several trials, but she did not remember what she had experienced in the previous meetings with Telenoid. In the Face condition she tended to describe the pleasure of her past in a vivid manner. The content of the conversation was only about her past, and not much about the speaker. She did not remember the recent news, and showed a gloomy look on her face when she talked about it. When interacting through Telenoid she seemed to consider that the robot was a child, then she became expressive and started talking aloud with Telenoid. When she talked to Telenoid, she asked about what it wanted to be in the future, displaying conversation fluency. She tended to physically interact with Telenoid by giving hugs and kisses, and touching head to head. Such physical behaviors were not found in the Face condition.

#### 3.1.2. Questionnaire

The questionnaire results are shown in Table 2. Comparing the averages from the Telenoid and Face conditions, we found significant differences in Sp_Q4 (Telenoid > Face, *t* = −2.75, *p* < 0.05), Ob_Q3 (Telenoid < Face, *t* = 3.28, *p* < 0.01), and Ob_Q4 (Telenoid > Face, *t* = −3.87, *p* < 0.01). Comparison between the first/last halves showed differences in Face condition's Sp_Q1 (*t* = −1.90, *p* < 0.10), Sp_Q6 (*t* = −2.14, *p* < 0.10), and Ob_Q1 (*t* = −2.13, *p* < 0.10). These results showed improvement in the communication in the later five trials.

**Table 2:** **Questionnaire results for trials with Ms. A**.

				**Telenoid**		**Face**	
		**Telenoid**	**Face**	**First half**	**Last half**	**First half**	**last half**
Speaker	Q1	3.6 (0.84)	4.1 (0.57)	3.8 (0.84)	3.4 (0.89)	3.8 (0.45)	4.4 (0.55)^*^
	Q2	3.6 (0.84)	3.7 (0.95)	3.6 (0.55)	3.6 (1.14)	3.6 (0.89)	3.8 (1.10)
	Q3	3.4 (0.70)	3.4 (0.70)	3.6 (0.55)	3.2 (0.84)	3.2 (0.84)	3.6 (0.55)
	Q4	4.4 (0.70)	3.6 (0.70)^**^	4.6 (0.55)	4.2 (0.84)	3.6 (0.55)	3.6 (0.89)
	Q5	3.7 (0.82)	3.7 (0.67)	3.6 (0.55)	3.8 (1.10)	3.4 (0.89)	4.0 (0.00)
	Q6	3.3 (1.25)	3.4 (0.70)	3.2 (1.10)	3.4 (1.52)	3.0 (0.71)	3.8 (0.45)^*^
	Q7	2.8 (0.63)	2.7 (0.95)	2.8 (0.45)	2.8 (0.84)	2.4 (1.14)	3.0 (0.71)
Observer	Q1	4.1 (0.74)	4.0 (0.82)	3.8 (0.84)	4.6 (0.55)	3.6 (0.55)	4.6 (0.89)^*^
	Q2	4.0 (0.94)	3.9 (0.57)	3.6 (1.14)	4.0 (1.22)	3.6 (0.55)	4.2 (1.30)
	Q3	3.1 (0.88)	3.8 (1.03) ^***^	2.8 (0.84)	3.6 (0.89)	3.6 (1.14)	4.0 (1.00)
	Q4	4.5 (0.71)	3.5 (0.85) ^***^	4.8 (0.45)	4.4 (0.55)	3.4 (1.14)	3.6 (1.14)
	Q5	4.0 (0.94)	3.6 (0.70)	4.2 (1.10)	3.4 (0.55)	3.2 (0.45)	3.4 (1.14)
	Q6	3.5 (1.08)	2.9 (0.57)	3.8 (0.84)	3.4 (1.14)	2.8 (0.45)	3.0 (0.71)
	Q7	3.3 (0.82)	3.5 (0.53)	3.4 (0.55)	3.2 (0.84)	3.2 (0.45)	3.4 (0.55)

Left column indicates overall comparison results between the Telenoid condition and the Face condition; Righthand two columns indicate first/latter half period summary for each condition. Values in the table indicates: mean score, SD (in parenthesis), t-test result where

* *p < 0.1*,

** *p < 0.05*,

*** *p < 0.01*.

#### 3.1.3. Video analysis

**Figure 3:**
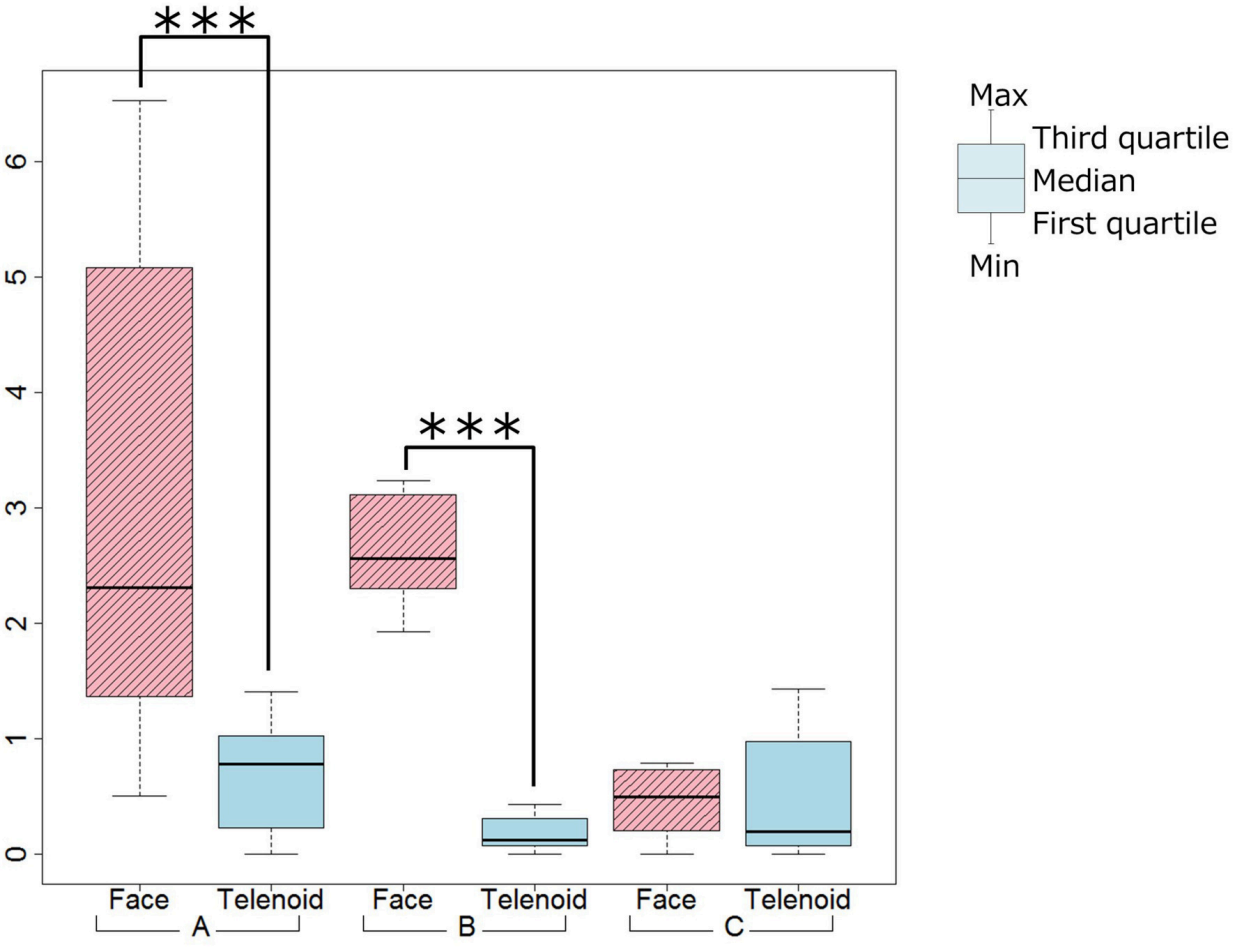
Gesture tendency (^*^p < 0.1, ^**^p < 0.05, ^***^p < 0.01).

**Figure 4:**
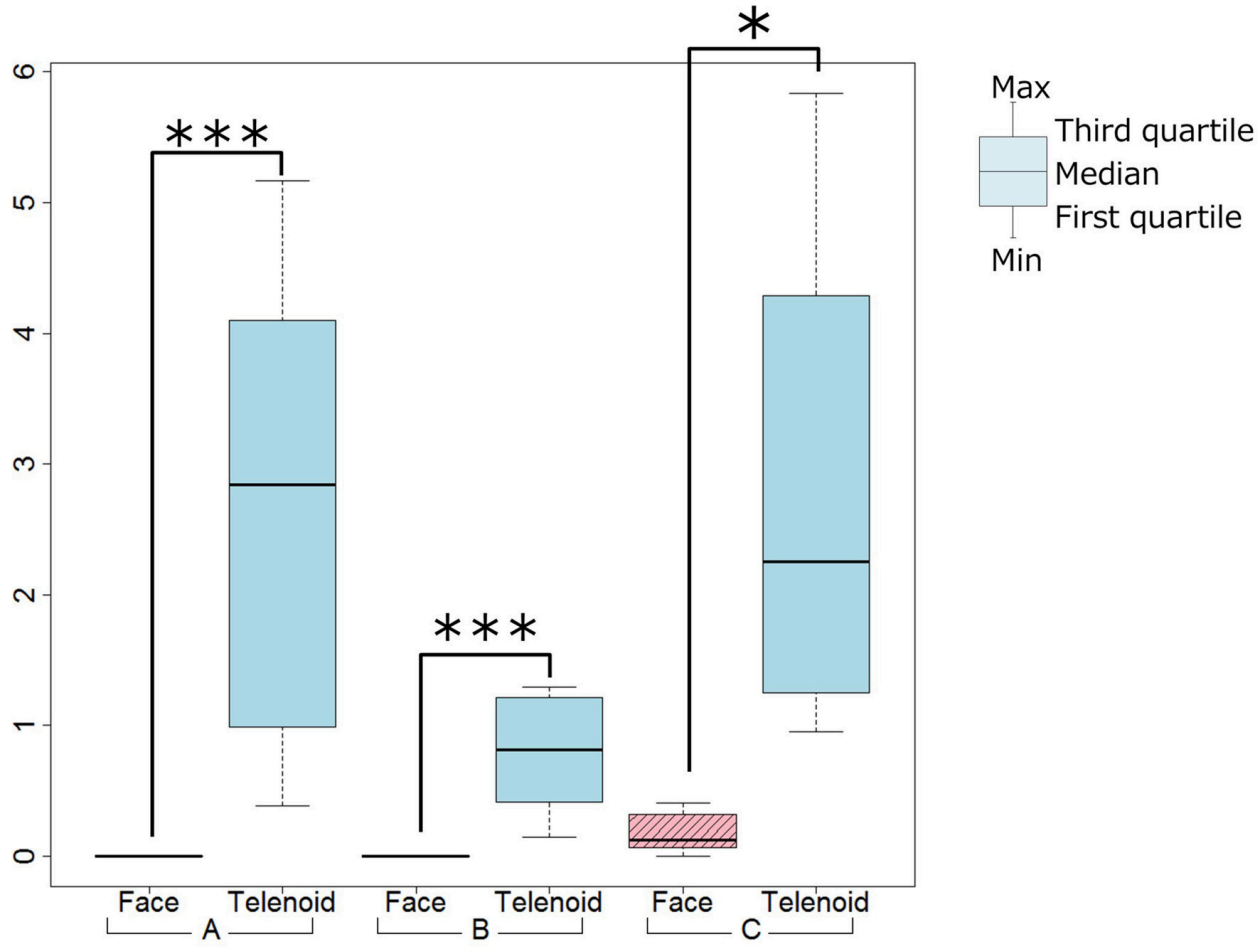
Physical contact tendency (^*^p < 0.1, ^**^p < 0.05, ^***^p < 0.01).

We used a paired *t*-test between the Telenoid and Face conditions and found significant differences for the frequency of gesture (Telenoid < Face, *t* = 3.75, *p* < 0.01), and the frequency of physical contact (Telenoid > Face, *t* = −5.40, *p* < 0.01; Figures 3, 4). We did not find significant differences for the frequency of gestures or physical contact between the first and last five trials.

### 3.2. Ms. B: 93 years old

#### 3.2.1. Diagnosis of dementia

Ms. B was diagnosed as AD in 2010. The test results for the diagnosis of dementia before and after the experiment are shown in Table 3.

**Table 3:** **Diagnosis of dementia test result of Ms. B**.

		**Before (9/4/2014)**	**After (3/26/2015)**
MMSE		17/30	14/30
QOL-D	Positive affect	28/28	20/28
	Negative affect and actions	12/24	10/24
	Ability of communication	20/20	20/20
	Restlessness	11/20	8/20
	Attachment with others	16/16	16/16
	Spontaneity and activity	16/16	16/16
DBD		25/112: Defect of memory	26/112: Defect of memory
		and fecal incontinence	and fecal incontinence
NPI-NH		None	Agitation/aggression
			Frequency 4, severity 1, caregiver distress 1
			Anxiety
			Frequency 4, severity 1, caregiver distress 2
BI		85/100	85/100
VTI		8/10	9/10

Ms. B had a gentle personality, but sometimes she rejected care and had problems with other residents and caregivers. She had severe episodic memory disorder and rarely remembered what she experienced in previous meetings with Telenoid and speakers. Her MMSE scores were 17 (before) and 14 (after), which indicates that she had moderate dementia. Mental and physical problems were rarely found by the tests, and she was generally calm during the experiments. She talked about herself in the Face condition, while asking more questions and making physical contact in the Telenoid condition.

#### 3.2.2. Questionnaire

The questionnaire results are shown in Table 4. Comparing the averages from the Telenoid and Face conditions, we found significant trends in Sp_Q4 (Telenoid > Face, *t* = −1.92, *p* < 0.10), Sp_Q6 (Telenoid > Face, *t* = −1.86, *p* < 0.10), Ob_Q1 (Telenoid > Face, *t* = −2.06, *p* < 0.10), Ob_Q4 (Telenoid > Face, *t* = −2.21, *p* < 0.10), and Ob_Q5 (Telenoid > Face, *t* = −2.23, *p* < 0.10). We also found significant differences in Ob_Q6 (Telenoid > Face, *t* = −2.69, *p* < 0.05). Comparison between the first/last halves showed significant trends in Telenoid condition's Ob_Q3 (*t* = −2.14, *p* < 0.10) and significant differences in Face condition's Sp_Q7 (*t* = −2.36, *p* < 0.05). These results showed improvement in communication in the last five trials.

**Table 4:** **Questionnaire results for trials with Ms. B**.

				**Telenoid**		**Face**	
		**Telenoid**	**Face**	**First half**	**Last half**	**First half**	**Last half**
Speaker	Q1	3.4 (1.07)	3.7 (0.82)	3.4 (1.14)	3.4 (1.14)	3.4 (0.89)	4.0 (0.71)
	Q2	3.5 (1.08)	3.5 (0.85)	3.6 (0.89)	3.4 (1.34)	3.2 (0.84)	3.8 (0.84)
	Q3	3.3 (0.67)	3.1 (0.74)	3.0 (0.71)	3.6 (0.55)	3.2 (0.84)	3.0 (0.71)
	Q4	4.1 (0.88)	3.3 (0.67)^*^	4.0 (0.71)	4.2 (1.10)	3.2 (0.45)	3.4 (0.89)
	Q5	2.9 (0.88)	3.1 (0.99)	3.0 (0.71)	2.8 (1.10)	3.0 (1.00)	3.2 (1.10)
	Q6	3.6 (0.84)	3.1 (0.88)^*^	3.4 (0.89)	3.8 (0.84)	3.2 (0.84)	3.0 (1.00)
	Q7	3.0 (0.82)	3.3 (0.82)	3.0 (1.00)	3.0 (0.71)	2.8 (0.84)	3.8 (0.45)^**^
Observer	Q1	4.0 (0.94)	3.2 (1.03)^*^	4.2 (1.10)	3.8 (0.84)	3.2 (0.84)	3.2 (1.30)
	Q2	3.7 (0.95)	3.4 (0.97)	3.8 (1.30)	3.6 (0.55)	3.2 (0.84)	3.6 (1.14)
	Q3	3.4 (0.70)	3.3 (0.67)	3.0 (0.71)	3.8 (0.45) ^*^	3.0 (0.00)	3.6 (0.89)
	Q4	4.1 (0.74)	3.2 (1.03)^*^	3.8 (0.84)	4.4 (0.55)	2.8 (0.84)	3.6 (1.14)
	Q5	3.7 (0.82)	2.9 (0.74)^*^	4.0 (0.71)	3.4 (0.89)	2.6 (0.55)	3.2 (0.84)
	Q6	3.5 (0.71)	2.8 (0.63)^**^	3.6 (0.89)	3.4 (0.55)	2.6 (0.55)	3.0 (0.71)
	Q7	3.1 (0.74)	3.3 (0.48)	2.8 (0.84)	3.4 (0.55)	3.2 (0.45)	3.4 (0.55)

Left column indicates overall comparison results between the Telenoid condition and the Face condition; Righthand two columns indicate first/latter half period summary for each condition. Values in the table indicates: mean score, SD (in parenthesis), t-test result where

* *p < 0.1*,

** *p < 0.05*,

^***^p < 0.01.

#### 3.2.3. Video analysis

We used a paired *t*-test between the Telenoid and Face conditions and found significant differences for the frequency of gestures (Telenoid < Face, *t* = 11.09, *p* < 0.01), and the frequency of physical contact (Telenoid > Face, *t* = −4.89, *p* < 0.01; Figures 3, 4). We did not find any significant differences for the frequency of gestures or physical contact between the first and last five trials.

### 3.3. Ms. C: 85 years old

#### 3.3.1. Diagnosis of dementia

Ms. C was diagnosed as AD in 2004. Her test results for the diagnosis of dementia before starting the experiments are shown in Table 5. Ms. C was transferred to a special nursing home for the elderly at the end of the experiment and could conduct the test after the experiment. Group home for the elderly with dementia is usually for the seniors with mild dementia, who need a little support to live by themselves. Ms. C was in the home because there was no spare room in the special nursing home at the beginning of the experiment. She moved to the special nursing home when there was a spare room.

**Table 5:** **Diagnosis of dementia test result of Ms. C**.

		**Before (9/4/2014)**
MMSE		0/30
QOL-D	Positive affect	23/28
	Negative affect and actions	15/24
	Ability of communication	6/20
	Restlessness	5/20
	Attachment with others	12/16
	Spontaneity and activity	6/16
DBD		22/112: Apathy, refusal, and incontinence were found
NPI-NH	Hallucinations	Frequency 4, severity 1, caregiver distress 0
	Agitation/aggression	Frequency 4, severity 1, caregiver distress 1
	Anxiety	Frequency 4, severity 1, caregiver distress 1
	Apathy	Frequency 4, severity 1, caregiver distress 0
	Disinhibition	Frequency 4, severity 1, caregiver distress 1
	Irritability	Frequency 4, severity 3, caregiver distress 1
	Aberrant motor behavior	Frequency 4, severity 1, caregiver distress 1
BI		45/100
VTI		8/10

Her MMSE score was 0, indicating severe dementia. She tended to make ambiguous statements and repeat the same phrases. Verbal communication was difficult with her; however, she did not often show a problematic BPSD, and the caregiver distress points were not high. She held eye contact in the Face condition; however, the content of her conversation was difficult to understand. Similar behavior was observed in the Telenoid condition. But she played peekaboo with Telenoid, suggesting that she thought she was interacting with a baby.

#### 3.3.2. Questionnaire

Since Ms. C was transferred to a special nursing home for the elderly at the end of the experiment, we could not measure the diagnosis of dementia after the experiment for Ms. C. However, the questionnaire result and video analysis result during the experiment is measured in a same way as Ms. A and Ms. B.

The questionnaire results are shown in Table 6. Comparing the averages from the Telenoid and Face conditions, we found significant differences in Sp_Q2 (Telenoid < Face, *t* = 2.45, *p* < 0.05). Comparison between the first/last halves showed significant differences in Face condition's Ob_Q1 (*t* = −2.75, *p* < 0.05), Ob_Q2 (*t* = −5.77, *p* < 0.01), and Ob_Q3 (*t* = −2.89, *p* < 0.05). These results showed improvement in the communication in the last five trials.

**Table 6:** **Questionnaire results for trials with Ms. C**.

				**Telenoid**		**Face**	
		**Telenoid**	**Face**	**First half**	**Last half**	**First half**	**Last half**
Speaker	Q1	2.2 (1.03)	2.8 (0.92)	2.4 (1.14)	2.0 (1.00)	2.8 (1.10)	2.8 (0.84)
	Q2	2.2 (1.14)	3.0 (1.05)^**^	2.4 (1.52)	2.0 (0.71)	2.8 (1.30)	3.2 (0.84)
	Q3	2.0 (0.94)	2.2 (0.92)	2.2 (1.30)	1.8 (0.45)	2.4 (1.14)	2.0 (0.71)
	Q4	3.8 (1.23)	3.6 (0.52)	3.4 (1.34)	4.2 (1.10)	3.6 (0.55)	3.6 (0.55)
	Q5	3.1 (0.88)	3.0 (0.94)	3.2 (0.84)	3.0 (1.00)	2.6 (0.89)	3.4 (0.89)
	Q6	2.7 (0.95)	2.2 (0.63)	2.6 (1.14)	2.8 (0.84)	2.0 (0.71)	2.4 (0.55)
	Q7	2.3 (0.95)	2.1 (0.99)	2.2 (1.30)	2.4 (0.55)	1.8 (0.84)	2.4 (1.14)
Observer	Q1	2.3 (0.95)	2.7 (1.06)	2.2 (0.84)	2.4 (1.14)	2.0 (1.00)	3.4 (0.55)^**^
	Q2	2.1 (0.88)	2.6 (1.17)	2.0 (1.00)	2.2 (0.84)	1.6 (0.55)	3.6 (0.55)^***^
	Q3	2.3 (1.06)	2.1 (0.74)	2.0 (1.00)	2.6 (1.14)	1.6 (0.55)	2.6 (0.55)^**^
	Q4	3.5 (0.85)	3.3 (0.95)	3.8 (0.84)	3.2 (0.84)	3.4 (1.14)	3.2 (0.84)
	Q5	2.9 (0.99)	2.7 (0.67)	3.0 (1.00)	2.8 (1.10)	2.6 (0.55)	2.8 (0.84)
	Q6	2.7 (0.82)	2.3 (0.67)	2.8 (0.84)	2.6 (0.89)	2.4 (0.55)	2.2 (0.84)
	Q7	2.4 (0.70)	2.6 (0.70)	2.6 (0.55)	2.2 (0.84)	2.6 (0.89)	2.6 (0.55)

Left column indicates overall comparison results between the Telenoid condition and the Face condition; Righthand two columns indicate first/latter half period summary for each condition. Values in the table indicates: mean score, SD (in parenthesis), t-test result where ^*^p < 0.1,

** *p < 0.05*,

*** *p < 0.01*.

#### 3.3.3. Video analysis

We used a paired *t*-test between the Telenoid and Face conditions and did not find significant differences for the frequency of gestures. However, we did find a significant trend in the frequency of physical contact (Telenoid > Face, *t* = −2.06, *p* < 0.10; Figures 3, 4). We also did not find significant differences in the frequency of gestures or physical contact between the first and last five trials.

## 4. Discussion

### 4.1. Ms. A

When we compared the scores between the Telenoid and Face conditions, both Q4s from the speakers and observers were significantly positive for the Telenoid condition (Table 2). This means that Ms. A showed a more positive reaction when talking to Telenoid than talking face-to-face. In the Telenoid condition, she changed her voice tone as if talking to a child. She seemed to treat Telenoid like a child, which allowed her to communicate in a more relaxed manner, leading to a positive Q4 score for the Telenoid condition. In fact, there were comments on the questionnaire. Immediately after she met Telenoid, she said, “You are so cute. I love you." Whereas in the face-to-face condition, even though she seemed nervous at the beginning of the interaction, she gradually managed to have a smooth conversation. We compared the questionnaire scores for the first and last five trials. In the Face condition, Sp_Q1, Sp_Q6, and Ob_Q1 had significant differences; they increased in the latter trials. The participant talked cheerfully with Telenoid from the beginning and did not have any significant differences between the first and latter five trials.

For Ob_Q3 (Quality of conversation), the Telenoid condition's score was negative compared with that in the Face condition. This might be because the participant recognized Telenoid as a child and the conversations content was playful. From the video analysis results, the participant tended to make physical contact in the Telenoid condition and used gestures in the Face condition. This indicates that she used physical interactions with Telenoid instead of verbal communication, as if taking care of a child. In fact, she tended to physically interact with Telenoid by hugs and kisses and touching its head. Such physical behaviors were not found in the Face condition.

### 4.2. Ms. B

When we compared the Telenoid and Face conditions, both Q4s and Q6s from the speakers and observers were significantly or marginally positive for the Telenoid condition (Table 4). The speakers also often adapted to the participants by changing their voice using a voice changer to sound more like a child.

The video analysis showed that in the Telenoid condition the participant made more physical contact, which was rarely observed in the Face condition. This was expected since physical interactions are usually only held among close relations. The speaker observed such interactions through the monitor, which might cause her to have better conversations with more emotional expressions. During the conversation, Ms. B seemed to interact with Telenoid as if it were a child, as in the case of Ms. A. Ms. B became calm when talking with Telenoid, which might explain the positive result in Q4 in the Telenoid condition. In fact, several questionnaire comments said that the participant seemed to become nervous at the beginning of the interaction in the Face condition with less eye contact, while conversely other comments said that the participant was relaxed and smiled more often to Telenoid.

We found that the emotional state of the speaker (Q6) became positive because the speaker experienced a more positive reaction from Ms. B through Telenoid. Telenoid affected the participant positively, resulting in a different quality of interaction, which the speaker enjoyed. Thus, Telenoid improved the conversation of both the participant and the speaker. There were also positive face-to-face conversations between Ms. B and the speaker; however, in the Telenoid condition the speaker observed the interactions from a third-person point of view, which allowed the speaker to participate in conversations objectively and have more positive feedback than in the Face condition.

### 4.3. Ms. C

When comparing the questionnaire scores for the first and last five trials, Face condition's Ob_Q1, Ob_Q2, and Ob_Q3 had significantly positive points for the latter half (Table 6). This suggests that the speaker adapted to the participant in the latter half, although it was difficult at the beginning.

Compared with the Face condition, Sp_Q2 (Amount of conversation) was significantly negative in the Telenoid condition. For the participant who had difficulty in the conversations, non-verbal information becomes more important. In the Telenoid condition, the speaker operating Telenoid only received limited information through the camera. The limited information may cause difficulty for the speaker during conversation, lowering scores. One of the speaker's comments on the questionnaire said, “Non-verbal information, like holding hands and eye contact, is important, but communicating this through Telenoid was difficult." There were no such comments by the observers.

In the video analysis, we found no significant differences between the Telenoid and Face conditions for the frequency of gesture tendency, while the frequency of physical contact was significantly higher for the Telenoid condition. This indicates that the participant was also attempting to have non-verbal communication with Telenoid, the same as in the Face condition. Therefore, the speaker's questionnaire scores might rise by improving the Telenoid operating system to support more non-verbal communication. The results also indicate that Telenoid might be a viable platform for communicating with seniors with severe dementia.

### 4.4. Overall discussion

All three participants tended to have more physical contact in the Telenoid condition. This result also implies that participants interacting with Telenoid were less nervous from the beginning of the conversation. They treated Telenoid as a child, which is huggable and easier to touch. Since it is huggable, they felt free to interact with it from the beginning.

The results suggest that because Telenoid has a physical presence, the elderly can hold it and they also like its child-like appearance. We believe such results cannot be seen by existing robots, including telepresence robots or Paro. To support communication by robots, especially for seniors with dementia, the robots appearance has to be in a form that the elderly can recognize and talk to at a relatively close distance that simplifies physical interaction. The close distance allows elderly to recognize a robot easily and enable to touch, which is important to establish a good relationship (Caris-Verhallen et al., 1999).

The Q4 scores (participant's impression) from both Ms. A and Ms. B supported the Telenoid condition. Ms. A and Ms. B tended to make more gestures in the Face condition. The reason might be because the participants had difficulty moving their upper body to make gestures while holding Telenoid.

Compared with Ms. A and Ms. B, since Ms. C has severe AD, verbal communication is more difficult with her. Ms. C tended to use more gestures in conversation and showed no significant differences in gesture tendency between the Telenoid and Face conditions.

## 5. Conclusion

We discussed the possibility of introducing a teleoperated robot into an elderly care house for long-term interaction. We compared two conversation conditions: face-to-face and using a teleoperated robot, Telenoid. Our experiment results showed that two participants with moderate AD had positive reactions from talking with Telenoid. The result supports the previous research about positive reaction of elderly using Telenoid (Yamazaki et al., 2014), and moreover, we found the result compared to face-to-face communication for long term.

The third participant had severe AD, and it was difficult to verbally communicate with her. However, she interacted with Telenoid using non-verbal communication in a way that resembled the face-to-face condition. Thus, we conclude that Telenoid may trigger positive emotions in residents with moderate AD and suggest the possibilities of non-verbal communication with residents with severe AD as well.

To introduce a robot to elderly care houses, caregivers must constantly use it and residents must be discouraged from losing interest in it. We compared the questionnaire results of the first and latter five trials and found significant differences or tendencies for five items in the Face condition and one item in the Telenoid condition, indicating that in the Face condition, people had better conversations as the experiment went on, and in the Telenoid condition, the quality of the conversation remained high. As for the face-to-face conversation, we believe this is because both the seniors and the speakers felt nervous at the beginning and took time to have effective conversations. On the other hand, people communicated smoothly through Telenoid from the beginning. The Telenoid condition had fewer items to improve in the latter five trials; however, no item worsened. This indicates that Telenoid did not lose the interest of the residents, not even at the end of the experiment. The robot we used in this study, Telenoid, is teleoperated and the operator can behave and speak in a variety of ways. Such nature of Telenoid may make it more alive, and interacting with Telenoid will likely to appear to be closer to human–human interaction than other robots such as Paro, QRIO, or Robovie as mentioned in the previous section. Since the state-of-art of artificial intelligence technology is quite limited, especially for having conversation with people, the teleoperation system used in Telenoid seems to be a very effective and practical solution.

The Telenoid users monitored the positive reactions of participants through a camera. The speaker may become motivated to better care for the patients by watching such interactions that cannot be seen in face-to-face communication. If caregivers were to use Telenoid, they might become emotionally expressive and enjoy conversations with seniors, boosting their motivation to care for those living with dementia. Observing the residents from a third-person point of view and communicating in a manner that is not possible face-to-face might improve caregiver attitudes, resulting in better relationships and an improved atmosphere in the facility. This could help caregivers and facility residents get to know each other better and eventually lower the turnover rate for the former.

If seniors suffer from severe AD, they rarely respond to care. As a result, caregivers have difficulty communicating with their charges and become discouraged. Observer's questionnaire result shows that the impression of residents with mild AD will become more cheerful when talking to Telenoid. This indicates that caregivers observing the interaction between Telenoid and the residents can notice the cheerful behavior of the residents, which might motivates caregivers. Also, if the caregiver met the resident for first time, the caregiver might have difficulty talking to the resident. By using Telenoid, the caregiver can easily have a conversation and understand the characteristics of the resident, which can be useful for the next meeting.

Caregivers sometimes have difficulty telling residents to do something. Residents sometimes refuse to wake up in the morning or eat lunch. Such refusal, which is caused by BPSD, can sometimes be solved by interacting with others. In such cases, Telenoid might be used as other people and interact with residents.

However, the experiment did not prove the effect of Telenoid itself, since the speakers had conversations both with Telenoid and face-to-face. Having conversations through Telenoid might reduce the nervousness of a speaker who is talking face-to-face, or the opposite effect might have happened. Although the speakers and residents experienced conversations in both forms, the Telenoid results showed significantly higher evaluations. Therefore, the Telenoid conversations outperformed the face-to-face conversations, but no cross effect are clear from the results here. We have to add a speaker-only condition using the Telenoid condition and only the face-to-face condition to reveal such an effect.

Another limitation of the current study lies in that its results do not show the effect of using Telenoid in comparison with other robots. We found positive results in the Telenoid conditions, perhaps not because of Telenoid, but since seniors with AD forgot the previous meetings. Future work has to include other robots and compare them to reveal long-term effects. So far we have only acquired a partial result with Telenoid because experimenters and volunteers were necessary for supporting the experiment. Caregivers had difficulty setting up Telenoid and using it properly since they were too busy with other tasks If the volunteers at the facility can operate Telenoid from their homes, the load of using it will decrease. We will consider a plan that introduces Telenoid and its appropriate usage in future work.

## Author contributions

SN and SS designed the experiment. KK, SN, and SS carried out the experiment at the care facility. KK and SS analyzed the results. KK and SN mainly prepared the manuscript.

### Conflict of interest statement

The authors declare that the research was conducted in the absence of any commercial or financial relationships that could be construed as a potential conflict of interest. The reviewer KB and handling Editor declared their shared affiliation, and the handling Editor states that the process nevertheless met the standards of a fair and objective review.
